# SOX2 Expression Does Not Guarantee Cancer Stem Cell-like Characteristics in Lung Adenocarcinoma

**DOI:** 10.3390/cells13030216

**Published:** 2024-01-24

**Authors:** Seung-Hyun Bae, Kyung Yong Lee, Suji Han, Chul Won Yun, ChanHyeok Park, Hyonchol Jang

**Affiliations:** 1 Division of Rare and Refractory Cancer, Research Institute, National Cancer Center, Goyang 10408, Republic of Korea; bsh8918@ncc.re.kr (S.-H.B.);; 2 Department of Cancer Biomedical Science, National Cancer Center Graduate School of Cancer Science and Policy, Goyang 10408, Republic of Korea; kylee@ncc.re.kr; 3 Division of Cancer Biology, Research Institute, National Cancer Center, Goyang 10408, Republic of Korea

**Keywords:** SOX2, cancer stem cell-like properties, CRISPR/Cas9, shRNA, lung adenocarcinoma

## Abstract

Effectively targeting cancer stemness is essential for successful cancer therapy. Recent studies have revealed that *SOX2*, a pluripotent stem cell factor, significantly contributes to cancer stem cell (CSC)-like characteristics closely associated with cancer malignancy. However, its contradictory impact on patient survival in specific cancer types, including lung adenocarcinoma (LUAD), underscores the need for more comprehensive research to clarify its functional effect on cancer stemness. In this study, we demonstrate that *SOX2* is not universally required for the regulation of CSC-like properties in LUAD. We generated *SOX2* knockouts in A549, H358, and HCC827 LUAD cells using the CRISPR/Cas9 system. Our results reveal unchanged CSC characteristics, including sustained proliferation, tumor sphere formation, invasion, migration, and therapy resistance, compared to normal cells. Conversely, *SOX2* knockdown using conditional shRNA targeting *SOX2*, significantly reduced CSC traits. However, these loss-of-function effects were not rescued by *SOX2* resistant to shRNA, underscoring the potential for SOX2 protein level-independent results in prior siRNA- or shRNA-based research. Ultimately, our findings demonstrate that *SOX2* is not absolutely essential in LUAD cancer cells. This emphasizes the necessity of considering cancer subtype-dependent and context-dependent factors when targeting *SOX2* overexpression as a potential therapeutic vulnerability in diverse cancers.

## 1. Introduction

Cancer stem cells (CSCs) are implicated in tumor progression, metastatic spread, and drug resistance [1,2]. These specialized cells play critical roles in tumor heterogeneity and in developing resistance to cancer treatment by influencing CSC-like properties such as growth, invasion, stemness, and therapy response across a wide range of cancer types [3,4,5,6]. Therefore, identifying key regulators of CSC-like properties provides opportunities to understand new mechanisms of cancer treatment.

*SOX2* (sex-determining region Y-box 2) was initially discovered as a transcription factor, playing an important role in maintaining the stemness of embryonic stem cells and inducing the transition of non-pluripotent cells to pluripotent stem cells [7,8]. Recent studies, however, demonstrate that an increase in its expression and gene amplification is frequently found in various cancer types and is clinically implicated in the poor prognosis of patients [9]. Abnormal expression of *SOX2* in adult differentiated cells has been significantly associated with the initiation and development of tumors [10,11,12]. These findings suggest that elevated expression of *SOX2* in diverse tumors plays a crucial role in cancer malignancy. Consequently, targeting *SOX2* emerges as a potential therapeutic strategy to enhance various cancer treatments.

However, several reports have claimed that *SOX2* can function as a tumor suppressor in certain cancers. It has been demonstrated that *SOX2* suppresses cell migration and the invasion of gastric cancers (GCs), and its low expression, frequently seen in GCs, is strongly associated with poor outcomes for GC patients [13]. In addition, there is a report suggesting that *SOX2* plays a negative role in colorectal cancers (CRCs) by affecting CSC-like properties and metastasis [14]. Taken together, these contradictory findings imply that studies unraveling the role of *SOX2* in regulating cancer traits should be executed with meticulous consideration of its oncogenic and onco-suppressive activities as well as its specificity to different cancer types [9,15].

Lung cancer, which ranks second in terms of cancer-related mortality, has two subtypes: small-cell lung carcinoma (SCLC) and non-small-cell lung carcinoma (NSCLC) [16]. It appears that conventional therapeutic approaches like chemotherapy and radiotherapy encounter resistance due to the presence of CSC, which contributes to the cancer heterogeneity and plasticity of NSCLC [17]. Lung adenocarcinoma (LUAD), one of the NSCLC subtypes, is the most prevalent and accounts for 60% of all NSCLC cases [18].

Previous reports have asserted that *SOX2* functions as a key regulator of CSC-like characteristics of LUAD. Multiple studies have shown that the reduction of *SOX2* expression using shRNA or siRNA leads to decreased proliferation and CSC-like characteristics in various LUAD cell lines [19,20,21,22]. However, these findings were derived from specific cell lines or through siRNA or shRNA methods, potentially introducing off-target activities. Additionally, conflicting reports exist regarding the correlation between *SOX2* expression and the survival of LUAD patients in different research groups. While Sholl et al. have reported a significant association between *SOX2* amplification (present in nearly 20% of LUAD cases) and poor prognosis in LUAD patients, Brcic et al. have contradicted this, stating that there is no significant correlation between the two [23,24]. Moreover, side population (SP) cells, identified as a subset of stem cells, exhibit *SOX2* expression, which, when overexpressed, subsequently enhances the tumorigenicity in 4 out of 9 LUAD cell lines, suggesting that the essential role of *SOX2* varies among different SP cell lines [25]. In essence, comprehensive research is necessary to unveil the role of *SOX2* in the malignancy of LUAD.

In this study, we generated knockout and conditional knockdown of *SOX2* in LUAD cell lines through the CRISPR/Cas9 (Cas9) and doxycycline-inducible shRNA techniques, respectively. Through multiple loss-of-function experiments using the complete knockout cells, we were able to provide evidence that *SOX2* is dispensable for CSC-like traits in LUAD cells, which contradicts previous findings [19,20,21,22]. Our sh*SOX2* cell lines with *SOX2* reconstitution strongly suggest a potential misinterpretation of previous findings based on transient knockdown using siRNA or shRNA. Therefore, our findings emphasize that various factors like cancer type and experimental context should be considered when unraveling the roles of stemness-regulating factors, including *SOX2*.

## 2. Materials and Methods

### 2.1. Cell Culture

A549, H23, H358, and HCC827 cells were obtained from the Korean Cell Line Bank (Seoul, Republic of Korea) and were cultured in RPMI-1640 medium (Hyclone, Logan, UT, USA). The medium was supplemented with 10% heat-inactivated fetal bovine serum (Hyclone), 1% penicillin-streptomycin (Invitrogen, Carlsbad, CA, USA), and 5 μg/mL Cellmaxin plus (GenDEPOT, Katy, TX, USA). The cells were maintained at 37 °C in a humidified incubator containing 5% CO_2_. The embryonal carcinoma cell line NCCIT and the kidney cell line HEK293FT were cultured as described previously [26,27]. Cell lines were authenticated and checked for mycoplasma at the Genomics Core Facility (National Cancer Center, Goyang, Republic of Korea). All cells were used within 20 passages after obtaining them from the Korean Cell Line Bank.

### 2.2. Plasmids, Mutagenesis and Generation of Stable Cell Lines

Guide RNA sequences targeting *SOX2* (5′–GCTCGCCATGCTATTGCCGC–3′) were inserted into the lentiCRISPR v2 vector (Addgene plasmid #52961), and shRNA sequences (5′–CAGCTCGCAGACCTACATGAA–3′) targeting *SOX2* were inserted into the Tet-pLKO-puro vector (Addgene plasmid #21915). For the generation of *SOX2*-reconstitution vector, human *SOX2* wild-type sequences were PCR-amplified and inserted into the pULTRA vector (plasmid #24129). shRNA-resistant *SOX2* was generated using site-directed mutagenesis involving the substitution of three genomic sequences: c.636C>T, c.639G>A, and c.642G>A.

To generate a *SOX2*-knockout cell line (g*SOX2*) and a *SOX2*-knockdown cell line (sh*SOX2*), lentivirus production and infection were carried out as described previously [27]. Briefly, lentiCRISPR v2 and Tet-pLKO vectors were transfected with packaging vectors in 293FT cells using polyethylenimine (Polysciences Inc., Warrington, PA, USA) for g*SOX2* and sh*SOX2*, respectively. Cells were infected with filtered lentiviruses in the presence of 0.8 μg/mL polybrene (Sigma-Aldrich, St Louis, MO, USA). After two days, the infected cells were selected with 1~3 μg/mL puromycin (InvivoGen, San Diego, CA, USA) over 4 days. Single-cell selection was avoided in order to maintain cell line heterogeneity, and consequently, some non-knockout cells may have been mixed in the case of g*SOX2*. *SOX2* knockout status was confirmed through Western blot and immunofluorescent staining. Doxycycline (1 μg/mL, Sigma-Aldrich) was administered every 48 h to induce *SOX2* knockdown through the Tet-pLKO system.

To generate a cell line stably expressing *SOX2* resistant to shRNA, the sh*SOX2* cells were transfected with pULTRA vector or pULTRA-SOX2. Finally, sh*SOX2*/Mock and sh*SOX2*/*SOX2* cells were sorted into high GFP-intensity cells by FACSAria (BD Biosciences, San Jose, CA, USA) at the Flow Cytometry Core Facility (National Cancer Center).

### 2.3. RNA Extraction and Semi-Quantitative Reverse Transcription-PCR (Semi-Quantitative RT-PCR)

The semi-quantitative RT-PCR was conducted as described previously with slight modifications [28]. Total RNA was extracted using a PURY RNA Plus kit (GenDEPOT) and was reverse transcribed into complementary DNA (cDNA) using the DiaStar^TM^ 2X RT Pre-mix kit (SolGent, Daejeon, Republic of Korea) according to the manufacturer’s instructions. The primer sequences used in this study were as follows: *SOX2*, forward primer 5′-TACCTCTTCCTCCCACTCCA-3′; reverse primer 5′-GGGCAGTGTGCCGTTAATG-3′ (175 bp transcript); *ACTB*, forward primer 5′-CAAGATCATTGCTCCTCCTG-3′; reverse primer 5′-GAAAGGGTGTAACGCAACTA-3′ (181 bp transcript).

### 2.4. Western Blot Analysis

Cells were washed twice with cold PBS and were then lysed in RIPA buffer (Thermo Fisher Scientific, Sunnyvale, CA, USA) containing a protease inhibitor cocktail (GenDEPOT) for 20 min on ice. The supernatants were collected after centrifugation at 13,000× *g* for 10 min. Immunoblotting was performed as previously described [29]. A rabbit anti-SOX2 monoclonal antibody (Cell Signaling Technology, Danvers, MA, USA) was used followed by incubation with goat anti-rabbit IgG-HRP (Thermo Fisher Scientific). To identify β-actin as a loading control, mouse anti-β-actin monoclonal antibody (Sigma-Aldrich) was used, followed by incubation with goat anti-mouse IgG-HRP (Thermo Fisher Scientific).

### 2.5. Flow Cytometry

A549, H23, and H358 cells were trypsinized and washed with PBS. The cells were then fixed by ice-cold methanol for 30 min. The blocking solution containing 5% normal goat serum was added for 1 h, followed by a brief wash with PBS. Then, cells were sequentially incubated with rabbit anti-SOX2 monoclonal antibody (Cell Signaling Technology) and goat anti-rabbit Alexa 488 (Invitrogen) for an hour each on ice. After staining, samples were analyzed using a FACS Verse Flow Cytometer (BD Biosciences) at the Flow Cytometry Core Facility (National Cancer Center). SOX2-positive cells were quantified using FlowJo ver. 10.7 software (Tree Star Inc., Ashland, OR, USA).

### 2.6. Immunocytochemistry

Immunocytochemistry was performed as previously described [27]. Samples were visualized using a Zeiss Axio Imager M2 fluorescence microscope system (Carl Zeiss, Jena, Germany). Primary (a rabbit anti-SOX2 monoclonal antibody (Cell Signaling Technology) and secondary (a goat anti-rabbit Alexa 594 (Invitrogen)) antibodies were sequentially incubated. After staining with DAPI for the nuclei, the fluorescence intensity was quantified using ZEN 3.4 software (Carl Zeiss). Data analysis and plotting were conducted using GraphPad Prism version 5.03 (GraphPad Software Inc., San Diego, CA, USA).

### 2.7. Proliferation Assay and Drug Sensitivity Assay

Proliferation assays using Sulforhodamine B (SRB) were performed as previously described [30]. Briefly, 1000 cells of A549 and 2000 cells of H358 were seeded in 96-well plates in quintuplicate. On days 1, 2, 3, and 4, the cultured cells were fixed with 33% TCA solution. After washing five times with PBS, the cells were stained with 0.4% (*w*/*v*) SRB dissolved in 1% acetic acid for 30 min, and washed with 1% (*v*/*v*) acetic acid. The plates were completely dried, and the dyes were solubilized with a 10 mM tris base solution (pH 10.5) for 30 min. The absorbance was measured at 515 nm using a SPECTRO Star Nano microplate reader (BMG LABTECH, Offenburg, Germany).

For the drug sensitivity assay, cells were seeded in 96-well plates and treated with cisplatin (Sigma-Aldrich) or paclitaxel (Tocris Cookson, Bristol, UK) for 72 h. Cell viability was measured by the SRB assay.

### 2.8. Clonogenic Assay

A total of 500 cells of A549 were grown on a 6-well plate for 7 days, and 2000 cells of H358 and HCC827 were grown for 14 days, respectively. Cells were gently washed with PBS and stained with 0.02% crystal violet working solution containing 1% methanol and 1% formaldehyde in PBS. The number of colonies was manually counted as previously described [26].

### 2.9. Tumor Sphere Formation

The tumor sphere culture medium was prepared as previously described [30]. A549, H358, and HCC827 cells were seeded at a density of 500 cells/well in a 96-well Clear Flat Bottom Ultra-Low Attachment Microplate (Corning Incorporated, Corning, NY, USA) with tumor sphere culture medium and 25 μL of the fresh medium was added twice a week. After two weeks, images of each well were analyzed using Cytation 3 (BioTek, Winooski, VT, USA). Tumor spheres in each cell line were counted when they reached the following diameter: A549, ≥150 μm; H358, ≥80 μm; HCC827, ≥100 μm. The experiments were independently replicated three times, with triplicate in each case.

### 2.10. Transwell Assays for Migration and Invasion

The invasion assay was performed as described previously with minor modifications [30]. Briefly, 5 × 10^4^ of A549 and HCC827 cells, or 3 × 10^5^ of H358 cells in serum-free RPMI medium were plated into the upper chambers of the cell culture inserts with a polycarbonate filter (24-well, 8-μm pore size; SPL Life Sciences, Pocheon, Republic of Korea). The inserts were pre-coated with 50 μL of diluted Matrigel (1:50 dilution in serum-free medium; Corning Incorporated) and the lower chamber was occupied with complete medium containing 20% FBS. After 48 h, the filters were washed, and cells that had shifted to the opposite side were stained with a Diff-Quik Staining Kit (Sysmex, Kobe, Japan). For the migration assay, an entirely identical procedure of the invasion assay was applied, excluding only the step of pre-coating the inserts with diluted Matrigel, to specifically observe cell migration. Each assay was repeated separately at least three times.

### 2.11. Alkaline Phosphatase (AP) Staining Assay

AP Staining assays were performed as described previously [26]. Briefly, cells were seeded at a density of 2000 cells per well in a 6-well plate and fixed with fixative solution (4% paraformaldehyde in PBS) after 7 days. After rinsing with PBS, naphthol/fast red violet staining solution was added to each well and the plates were incubated in the dark at room temperature for 15 min. The wells were then rinsed with TBST (20 mM Tris-HCl, pH 7.4, 0.15 M NaCl, 0.05% Tween-20). The cells were covered with PBS to prevent drying, and the staining pattern was observed under an inverted light microscope.

### 2.12. Statistical Analysis

Statistical analysis was performed as previously reported [29]. Briefly, data were presented as means ± SD, and GraphPad Prism version 5.03 (GraphPad Software Inc., USA) was used to carry out a Student’s *t*-test with a *p*-value. All data were obtained from at least three independent experiments.

## 3. Results

### 3.1. Inter- and Intra-Cellular Heterogeneity in LUAD Cell Lines Regarding SOX2 Expression

**Figure 1:**
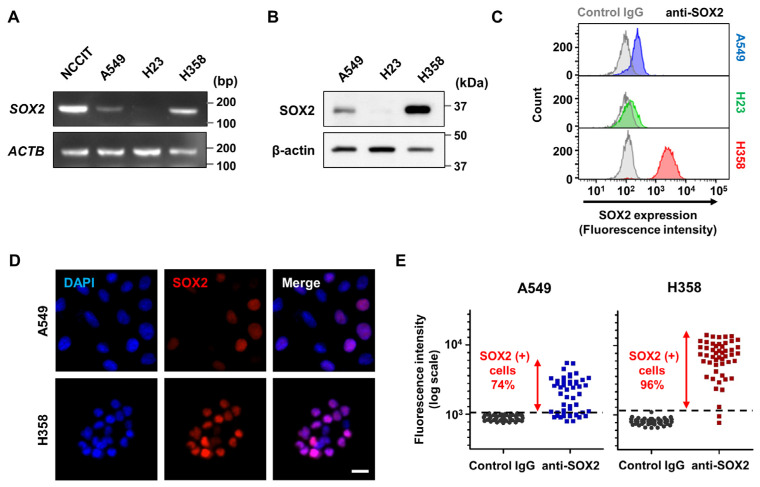
Inter- and intra-cellular heterogeneity in Lung adenocarcinoma (LUAD) cell lines regarding *SOX2* expression. (**A**) *SOX2* mRNA expression in LUAD cells. Semi-quantitative RT-PCR was conducted to analyze the mRNA expression of *SOX2* and *ACTB* (β-actin) using total RNA extracted from LUAD cells A549, H23, and H358. NCCIT, an embryonic carcinoma cell line, was used as a positive control. (**B**) SOX2 protein expression in LUAD cells. The antibodies mentioned were employed in Western blotting, with β-actin serving as a loading control. (**C**) Flow cytometry (FACSVerse) was utilized to assess SOX2 protein expression in individual LUAD cells, employing an anti-SOX2 antibody and secondary goat anti-rabbit Alexa 488. A Rabbit IgG was used as a negative control. (**D**) Representative images of immunofluorescence staining with anti-SOX2 antibody in A549 and H358 cells. Nuclei stained with DAPI. Scale bar: 20 μm. (**E**) Quantification of SOX2-positive cells using immunostaining. Cells labeled with SOX2, exhibiting fluorescence intensity surpassing that of the highest controls, were considered as SOX2-positive cells. *n* = 50 for each sample.

Cancer stem cell (CSC)-like properties have been demonstrated to have significant clinical implications in cancer malignancy, and therefore, the exploration of the associated key pathways has the potential to play a crucial role in deriving innovative cancer therapies targeting these properties. An essential modulator of stemness, *SOX2*, has been consistently identified as a promoting factor for CSC-like properties in diverse cancer types including LUAD, but conflicting findings have been reported concerning the impact of *SOX2* on patient survival in LUAD [9,24]. Since previous studies have consistently employed A549 as the LUAD cell line to establish the essential role of *SOX2* in CSC or CSC-like properties [19,21,22,23,26], our objective in this study was to investigate the correlation of *SOX2* expression with LUAD malignancy by examining several representative LUAD cell lines in addition to A549. To assess *SOX2* expression in LUAD cells, we analyzed the mRNA levels of *SOX2* in four LUAD cell lines: A549, H23, H358, and HCC827 using the Cancer Cell Line Encyclopedia (CCLE) through the DepMap portal. An embryonic carcinoma cell line, NCCIT, was also examined as a positive control for *SOX2* expression (Figure S1A). Although *SOX2* expression in LUAD cells was not comparable to that of NCCIT, a substantial amount of *SOX2* mRNA was observed in the following order: H358, A549, HCC827, and H23 cells (Figure S1A). Semi-quantitative RT-PCR and Western blotting analyses to measure both mRNA and protein levels of *SOX2* in some of the cell lines confirmed these results (Figure 1A,B and Figure S1B). While *SOX2* mRNA and protein expression were nearly undetectable in H23 cells, a substantial amount of *SOX2* mRNA and protein was observed in A549, H358, and HCC827 cells (Figure 1A,B and Figure S1B). As a result, we observed intercellular heterogeneity within the LUAD cell lines. While it is not always the case, CSCs are often a minority within the overall cancer cell population. Therefore, we aimed to determine the percentage of cells expressing SOX2 within a single LUAD cell line. To achieve this, we assessed the expression levels of SOX2 in individual cells using flow cytometry and immunocytochemistry (Figure 1C–E). Unlike H23, which showed minimal expression of SOX2, the majority of both A549 and H358 cell populations exhibited higher fluorescence intensity compared to the control, indicating a prevalence of cells with SOX2 expression in both LUAD cell lines (Figure 1C). Our immunocytochemistry results further confirmed that the majority of A549 and H358 cells expressed the SOX2 protein (74% of A549 cells and 96% of H358 cells). Interestingly, we observed heterogeneous patterns of SOX2 expression even among the SOX2-positive cell populations in both LUAD cell lines, as depicted in Figure 1D,E. Despite the heterogeneous patterns across the cell population, these findings highlight the presence of inter- and intra-cellular heterogeneity in SOX2 expression among various LUAD cells.

### 3.2. SOX2 Knockout Does Not Perturb Cell Proliferation of LUAD

**Figure 2:**
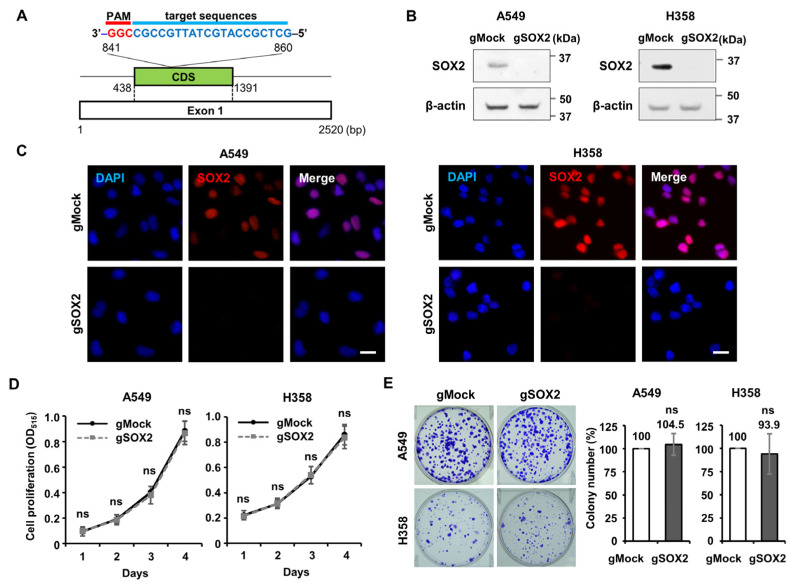
*SOX2* knockout does not perturb the cell proliferation of LUAD. (**A**) A schematic representation illustrating the g*SOX2* target site on *SOX2* Exon1 for the generation of *SOX2* knockout LUAD cell lines using the CRISPR/Cas9 system. (**B**) Immunoblots showing the SOX2 protein level in A549 and H358 cells stably expressing gMock or gSOX2. (**C**) Immunocytochemical analysis using anti-SOX2 antibody in Mock and SOX2 knockout LUAD cells. Scale bar: 20 μm. (**D**) No impact of *SOX2* knockout on the cell proliferation of LUAD cells. Cellular proliferation was measured using sulforhodamine B (SRB) assays, and each value was presented as optical density at 515 nm (OD_515_). ns, not significant. (**E**) No difference in the ability of a single cell to grow into a colony in LUAD *SOX2* knockout cells. Representative images (left) and the quantification (right) of relative colony number in each sample are shown. The crystal violet staining was performed 7 days after seeding of A549 cells and 14 days for H358 cells. Values represent means ± SD from three independent experiments. ns, not significant.

As mentioned in the introduction, a few studies have reported that *SOX2* knockdown suppresses the CSC characteristics in LUAD cells [19,20,21,22]. Given the contradictory prognostic impact of *SOX2* expression reported in some cancer types, we wondered about its knockout effect on proliferation and characteristics in LUAD cells. Therefore, we generated CRISPR/Cas9-mediated *SOX2* knockout in LUAD A549, H358, and HCC827 cells. A guide RNA targeting exon1 near the N-terminus of the *SOX2* coding sequence (CDS) (g*SOX2*) was expressed together with Cas9 in the LUAD cell lines (Figure 2A). Importantly, we used a heterogeneous cell population with *SOX2* knockout in our subsequent experiments, rather than knockout cells derived from single clonal selection, to avoid bias due to the selection dependency.

As expected, Western blotting and immunocytochemistry analyses verified that SOX2 expression was completely abolished in A549, H358, and HCC827 cells (Figure 2B,C and Figure S1C). Using these cell lines, we investigated whether *SOX2* knockout had any effect on LUAD proliferation using SRB and clonogenic assays to assess short-term growth rates and the ability of a single cell to grow into a colony, respectively (Figure 2D,E and Figure S1D). Surprisingly, we observed that *SOX2*-knockout LUAD cells of A549 and H358 showed no difference in proliferation compared to corresponding control cells (gMock) in the SRB assays (Figure 2D). Furthermore, even in clonogenic assay, no alteration in colony forming efficiency was observed due to *SOX2* knockout in A549, H358, and HCC827 cells (Figure 2E and Figure S1D). Meanwhile, *SOX2* knockout in an embryonic carcinoma cell, NCCIT, with the same g*SOX2*, resulted in a reduced self-renewal ability, as assessed by the alkaline phosphatase staining assay (Figure S2A,B), suggesting that g*SOX2* functionally affects the stemness of embryonic carcinoma cells. In conclusion, we demonstrated that *SOX2* knockout has no effect on the growth of certain LUAD cell lines, including A549 cells, which had been used to show SOX2′s essentiality for LUAD cell growth in previous studies.

### 3.3. SOX2 Is Not Required for Tumor Sphere Formation, Migration, and Invasion of LUAD Cells

**Figure 3:**
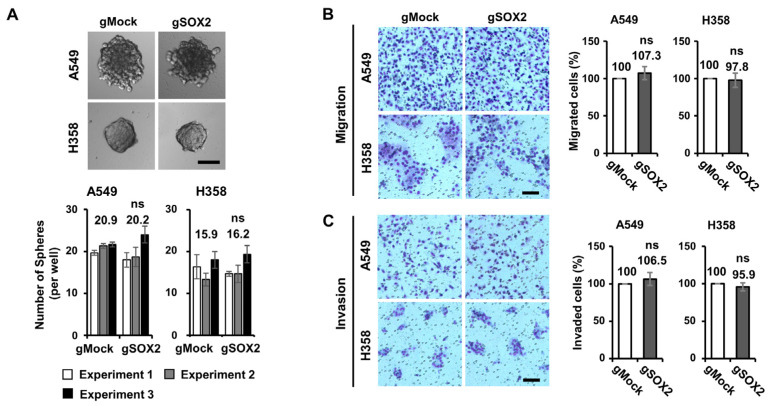
*SOX2* is not required for tumor sphere formation, invasion, and migration of certain LUAD cells. (**A**) No impact of *SOX2* knockout on tumor sphere (TS) formation in LUAD cells. TS formation assays were conducted 14 days post-seeding using A549 and H358 cells stably expressing gMock or g*SOX2*. Representative images (top) and the quantification of TS number (bottom) are shown. Results from each independent experiment (Experiment 1, 2 and 3) are presented as means ± SD (n = 3). ns, not significant. Scale bar: 100 μm. (**B**,**C**) No impact of *SOX2* knockout on the migration and invasion of LUAD cells. Migration (**B**) and invasion (**C**) assays were carried out using Mock and *SOX2* knockout A549 and H358 cells. Representative images (left) are shown, and the total number of migrated and invaded cells was normalized to that of gMock. Mean ± SD from three independent experiments are presented. Scale bar: 100 μm. ns, not significant.

Tumor sphere (TS) formation is often used to assess CSC-like characteristics, and specifically, it has been reported that *SOX2* overexpression enhances TS formation in H358 LUAD cells [31,32]. To determine the impact of *SOX2* on TS formation in LUAD, TS formation assays were conducted using *SOX2* knockout LUAD cell lines (Figure 3A and Figure S1E). While A549, H358, and HCC827 gMock successfully grew as spheres in a 3D culture, the corresponding *SOX2* knockout cells exhibited no discernible difference (Figure 3A and Figure S1E).

Additionally, our migration assays measuring cell movement, revealed that *SOX2* knockout had no impact on the migratory capacities of A549 and H358 LUAD cells (Figure 3B). In the invasion assay, which measures the penetrating ability of cells through physical barriers, we found that the invasive properties of these LUAD cells were not altered by *SOX2* knockout (Figure 3C). However, in the case of HCC827, *SOX2* knockout increased invasion (Figure S1F), suggesting the context-dependent variable roles of *SOX2*. Nonetheless, we were not able to find any evidence to show that *SOX2* promotes the migration and invasion of these LUAD cells. These results demonstrate that loss-of-function and gain-of-function phenotypes can be inconsistent even within the same cell line, highlighting the context-dependent role of *SOX2* in LUAD.

Overall, our findings suggest that *SOX2* is not essential for representative CSC-like traits such as TS formation, migration, and invasion.

### 3.4. shRNA-Resistant SOX2 Is Unable to Rescue Impaired Proliferation by shSOX2 in LUAD

**Figure 4:**
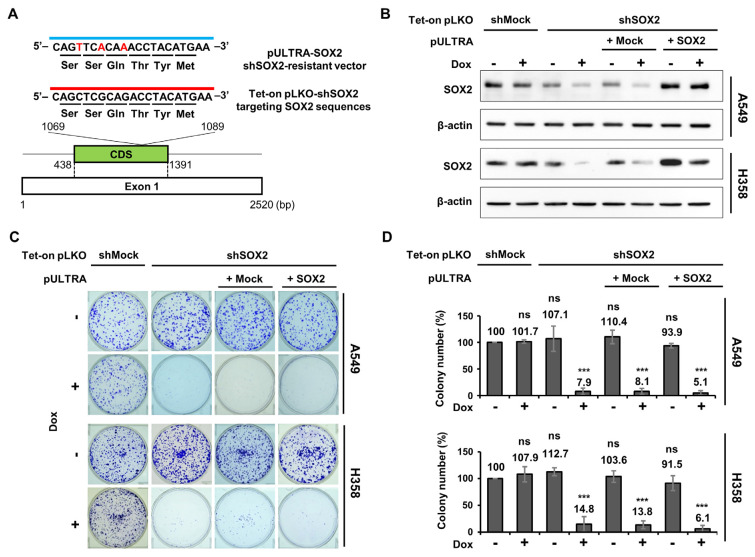
shRNA−resistant *SOX2* is unable to rescue impaired proliferation by sh*SOX2* in LUAD. (**A**) A schematic showing the target site for *SOX2* knockdown, with DNA sequence substitutions for shRNA-resistant *SOX2*. The target site (red line) was used to generate Tet-inducible *SOX2* knockdown (sh*SOX2*) in A549 and H358 cells. Red letters indicate three synonymous substitutions, reconstituting shRNA-resistant *SOX2* in sh*SOX2* cell lines (sh*SOX2*/+*SOX2*). (**B**) *SOX2* knockdown and its reconstitution in sh*SOX2* LUAD cell lines were assessed. Cells were treated with 1 μg/mL of _doxycycline_ (Dox) for 4 days, and SOX2 protein level was accessed by Western blotting. shMock and shMock + Mock were used as negative controls. (**C**,**D**) Reduced cell survival caused by sh*SOX2* is not restored by *SOX2* reconstitution in LUAD cells. Clonogenic assays were conducted using indicated A549 and H358 cell lines. Representative images (**C**) and the quantification (**D**) are shown. The colony number of each sample was normalized to that of shMock without Dox. Mean ± SD from three independent experiments. ***, *p* < 0.001; ns, not significant. *p* values were compared with shMock without Dox.

Given that conflicting outcomes have been reported in previous studies relying on siRNA- or shRNA-mediated down-regulation [19,25,33], we sought to determine what made these variations. To address this, we decided to establish shRNA-based *SOX2* knockdown (sh*SOX2*) cell lines in the same LUAD cells used for our knockout experiments. These cell lines were created in both A549 and H358 cells using validated sh*SOX2* from previous studies [34,35,36,37]. Additionally, sh*SOX2*-resistant *SOX2* was reconstituted in the sh*SOX2* cells (Figure 4A).

Following doxycycline (Dox) treatment for 4 days, *SOX2* knockdown (sh*SOX2*) in A549 and H358 cells resulted in a significant reduction of *SOX2* expression compared with control cells (shMock), while shRNA-resistant *SOX2* was effectively reintroduced in sh*SOX2* cells (sh*SOX2*/+*SOX2*) (Figure 4B).

In clonogenic assays using these cell lines, we found that the ability of a single cell to grow into a colony in sh*SOX2*/+*SOX2* cells showed no difference compared to shMock cells under conditions without Dox treatment (Figure 4C,D). Considering that *SOX2* expression in sh*SOX2*/+*SOX2* was higher than endogenous levels of A549 and H358 cells (lane 1 vs. lane 7 in Figure 4B), this finding suggests that *SOX2* overexpression does not impact LUAD growth. Notably, sh*SOX2* cells exhibited a significant reduction in clonogenicity compared to the shMock cells in the presence of Dox (Dox+), consistent with previous findings [19,25,33]. However, *SOX2* reconstitution was unable to reverse the growth retardation observed in A549 and H358 cells where endogenous *SOX2* was depleted by sh*SOX2* (Figure 4C,D). These results strongly indicate that growth impairment in LUAD cells observed with sh*SOX2* is not solely due to SOX2 protein downregulation.

### 3.5. SOX2 Expression Does Not Significantly Affect CSC-like Properties in LUAD Cell Lines

**Figure 5:**
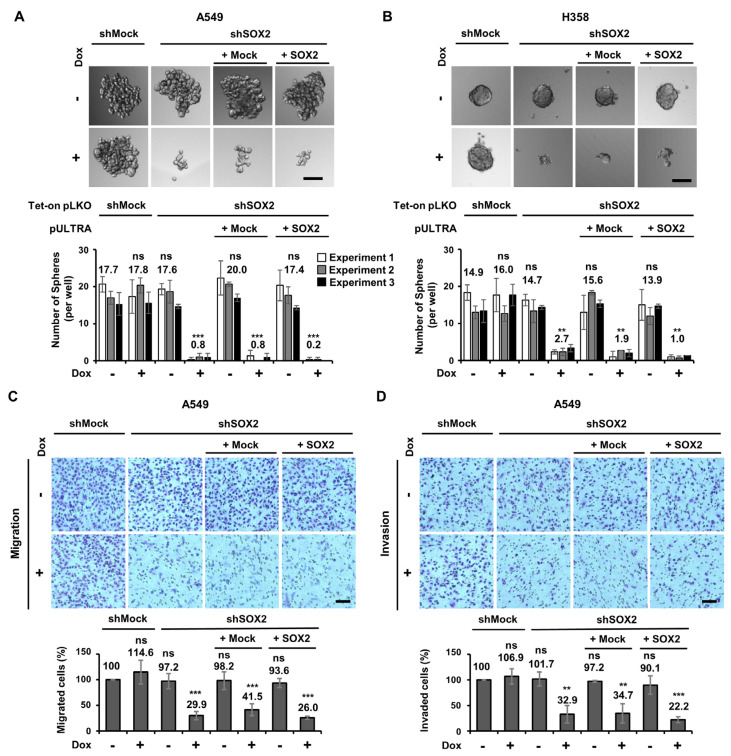
shRNA−resistant *SOX2* is unable to rescue suppression of sphere formation, invasion, and migration observed in sh*SOX2* LUAD cells. (**A**,**B**) The impaired sphere formation caused by sh*SOX2* is not restored by the reconstitution of *SOX2* in LUAD cells. ‘+’ Dox indicates that 1 μg/mL Dox was treated. Representative images (top) and the quantification of TS number (bottom) are shown. TS formation assays were conducted using shMock, sh*SOX2*, sh*SOX2*/+Mock, and sh*SOX2*/+*SOX2* in A549 (**A**) and H358 (**B**) cells. Results from each independent experiment (Experiment 1,2 and 3) are presented as means ± SD (n = 3). (**C**,**D**) Reconstitution of *SOX2* is unable to restore the reduced migration and invasion observed in sh*SOX2* LUAD cells. Migration (**C**) and invasion (**D**) assays using indicated A549 with or without Dox treatment. Means ± SD from three independent experiments are presented. ***, *p* < 0.001; **, *p* < 0.01 when compared with the shMock without Dox; ns, not significant; TS, tumor sphere. Scale bar: 100 μm.

Since *SOX2* knockout in LUAD cells did not influence TS formation, migration, and invasion (Figure 3), we next examined the impact of sh*SOX2*-mediated knockdown and its subsequent reconstitution on these CSC-like properties. Notably, the number of TS formations significantly decreased in both A549 and H358 cells having shRNA-mediated *SOX2* knockdown, and this reduction was not restored by *SOX2* reconstitution (Figure 5A,B). Moreover, impaired migration and invasion were observed in sh*SOX2* A549 cells compared to shMock cells, but these properties were not able to be rescued by *SOX2* reconstitution (Figure 5C,D). Overall, our findings consistently suggest that the effect of sh*SOX2*-mediated knockdown on CSC-like properties in LUAD cells might not be attributed to the downregulation of SOX2 protein level.

**Figure 6:**
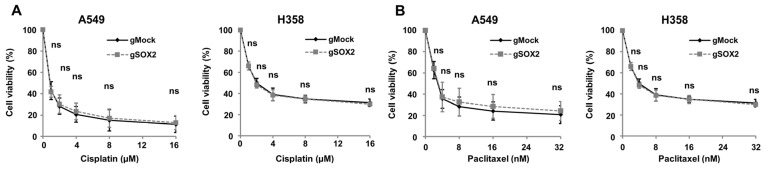
*SOX2* expression does not significantly affect cancer stem cell-like properties in certain LUAD cell lines. (**A**,**B**) Cellular response to chemotherapeutic drugs is not altered by *SOX2* knockout in LUAD cells. Indicated concentrations of cisplatin (**A**) or paclitaxel (**B**) were treated for 72 h in A549 and H358 cells stably expressing gMock and g*SOX2*. Cell viability was measured by SRB assay, and the values are presented as the mean ± SD from three independent experiments. ns, not significant.

CSC-like properties confer resistance to cancer drugs, often leading to relapse [38,39]. Since it has been reported that the overexpression of *SOX2* renders A549 cells resistant to cancer drugs, cisplatin and paclitaxel [22,40], we performed drug sensitivity assays of gMock and g*SOX2* LUAD cells using the cancer drugs (Figure 6). However, *SOX2* knockout in both A549 and H358 cells did not lead to an increase of cellular sensitivity to cisplatin and paclitaxel compared to control cells (Figure 6A,B). These results indicate that there are LUAD cells where *SOX2* is not essential for drug resistance.

## 4. Discussion

While multiple studies have identified *SOX2* as a crucial factor in controlling CSC-like properties in various cancer types [12], the precise role of *SOX2* in specific cancers remains controversial. In this study, we have demonstrated that the indispensability of *SOX2* for CSC-like characteristics is not absolute in all LUAD. Neither knockout nor overexpression of *SOX2* in LUAD cells resulted in any changes in growth, sphere formation, migration, invasion, or resistance to therapeutic drugs. In general, the CSC model is based on the concept that a small subset within bulk tumors initiates cancer progression and relapse [41]. Our finding that SOX2 protein is substantially expressed in numerous individual cells of LUAD suggests the possibility that it might not function as a biomarker for CSCs. Although *SOX2* expression tends to have an unfavorable impact on patient prognosis in the majority of cancer types, it is also linked to a favorable prognosis in at least four types of cancer including gastric, head and neck squamous carcinoma, lung SCC, and ovarian cancers (as reviewed in [9]). Consequently, our findings highlighting its dispensable role in LUAD cells emphasize the potential for *SOX2* to exhibit different functions varying according to the cancer type or context.

Previous reports have documented conflicting functional effects of *SOX2*, even within identical cancer types [42,43,44,45]. In LUAD, there is a report showing that *SOX2* promotes the growth of spheroids and confers cancer drug resistance in A549 cells [44]. Conversely, it has also been documented that *SOX2* expression enhances sensitivity to tyrosine kinase inhibitors in EGFR-mutated LUAD patients [45]. These studies have a common aspect in obtaining loss-of-function outcomes using RNA interference (RNAi)-mediated techniques. CRISPR and RNAi are commonly employed in loss-of-function studies and, unfortunately, share concerns about on-target efficacy. However, a growing body of evidence indicates that RNAi has more pervasive off-target effects than CRISPR based on experiments, screenings, and computational methodologies [46,47,48]. This can be attributed in part to partial complementation between siRNAs and off-target RNAs, which can lead to unexpected silencing even with sequences as short as 10 base pairs. Additionally, exogenous shRNAs may competitively occupy the RNA-induced silencing complex (RISC) instead of endogenous microRNAs [49,50]. Furthermore, a previous report has highlighted the risks associated with using non-targeting shRNA controls for functional studies. Specifically, it was found that *SHC016*, one of the non-targeting shRNA controls in the MISSION library, caused the unintended silencing of small nuclear ribonucleoprotein *Sm D3 (SNRPD3)*, resulting in deleterious effects in human and murine cell lines [51]. Therefore, when interpreting the results of RNAi-based loss-of-function studies, it is crucial to validate that the observed effects are indeed due to changes in the target protein by conducting target protein restoration experiments.

We demonstrated that the sh*SOX2*-mediated phenotype was not restored by *SOX2* reconstitution, suggesting that previous results from shRNA- or siRNA-mediated *SOX2* loss-of-function experiments may not be solely attributed to changes in SOX2 protein levels. One key difference between RNAi-based knockdown and CRISPR knockout is that RNAi reduces the mRNA of the target protein, while CRISPR does not. Considering that RNA plays various independent roles beyond serving as an intermediate product for protein translation, the different outputs observed in CRISPR and RNAi-based loss-of-function experiments may be attributed to differences at the mRNA level. Therefore, previous concepts regarding the role of *SOX2* in the stemness of various cancers, primarily derived from shRNA- or siRNA-mediated approaches, need to be reconsidered with caution.

Intriguingly, *SOX2* knockout melanoma cells using the CRISPR/Cas9 system also displayed similar tumor growth patterns compared to the control in xenotransplantation experiments [43]. In other words, the non-essential role of *SOX2* has also been found in melanoma, raising the possibility that there might be other cancer types where *SOX2* does not function as well. So, the next question would be why *SOX2* does not play a role in certain cancer types. The group B Sox proteins, to which *Sox2* belongs, are functionally similar and divided into two subgroups, including transcriptional activating and repressing subgroups [52,53]. Of note, it has been reported that there is functional overlap among subgroup members, suggesting that there might be compensation among these family components in *SOX2* knockout cells. In addition, variable functions of *SOX2*, regulated by factors such as post-translational modifications (PTMs) could lead to diverse patterns in various cancer types. Therefore, future studies should prioritize a more comprehensive analysis that considers contextual factors and the specific characteristics of distinct cancer types.

## 5. Conclusions

In conclusion, our study indicates that not all LUAD requires the SOX2 protein for CSC-like properties, including proliferation, sphere formation, invasion, migration, and sensitivity to chemotherapeutics. Our CRISPR/Cas9-mediated *SOX2* knockout demonstrates that there is no functional connection between SOX2 expression and various CSC-like and EMT traits, at least in a few cell lines of LUAD. While these findings challenge the conventional notion of *SOX2*’s indispensability in various cancer types, it is important to acknowledge and consider the potential cancer subtype-specific and context-specific role of *SOX2*, which can manifest differently in various types of cancers. Therefore, comprehensive analysis is required for future studies on the significance of *SOX2* as a potential target for innovative cancer treatments in controlling the maintenance, progression, and stemness of LUAD.

## Data Availability

Data are contained within the article.

## Supplementary Materials

The following supporting information can be downloaded at: https://www.mdpi.com/article/10.3390/cells13030216/s1, Figure S1: *SOX2* mRNA expression in LUAD cells from a public database and lack of suppression in *SOX2* knockout for cell proliferation, tumor sphere formation, and invasion of HCC827 cells. Figure S2: *SOX2* knockout reduces self-renewal ability of embryonic carcinoma cells, NCCIT.
