# Supplementary material for: SOX2 Expression Does Not Guarantee Cancer Stem Cell-like Characteristics in Lung Adenocarcinoma
Source: Cells. 2024 Jan 24;13(3):216. doi: 10.3390/cells13030216 (PMC10854781; doi:10.3390/cells13030216)
Supplement: Supplementary file 1 [file cells-13-00216-s001.zip › cells-2751159-supplementary.pdf]

Supplementary Materials

Title: SOX2 Expression Does Not Guarantee Cancer Stem Cell-like Characteristics in Lung Adenocarcinoma.

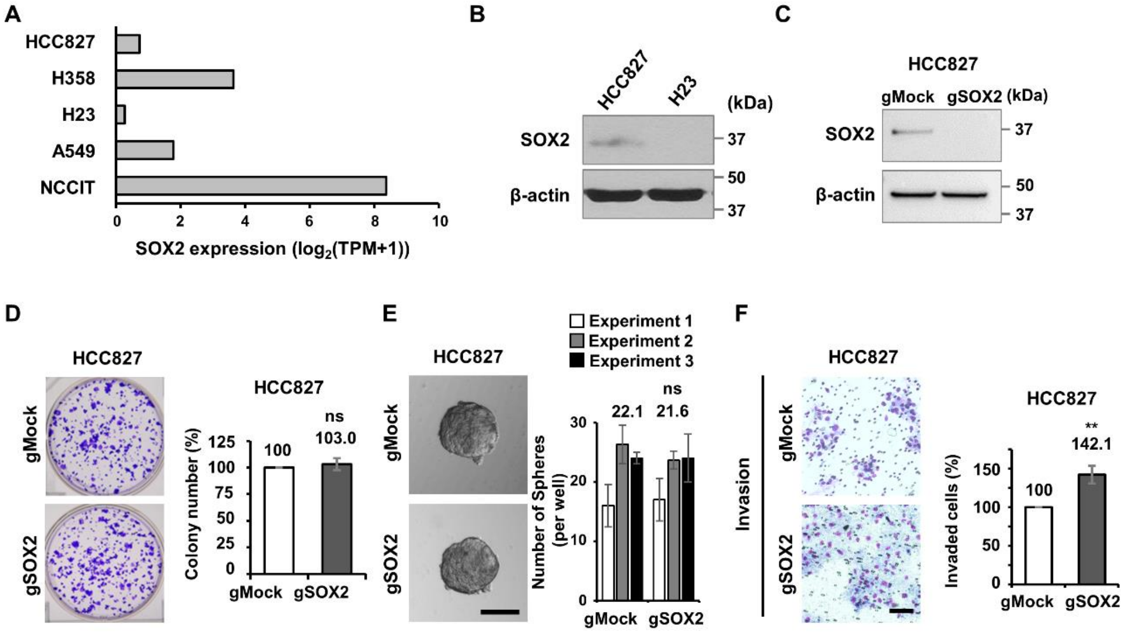

Figure S1. *SOX2* mRNA expression in LUAD cells from a public database and lack of suppression in *SOX2* knockout for cell proliferation, tumor sphere formation, and invasion of HCC827 cells. (A) SOX2 mRNA expression data in indicated cell lines (CCLE). Corresponding gene expression (log2(TPM + 1)) was downloaded from the DepMap portal (https://depmap.org/portal). (B) Immunoblots for the comparison of SOX2 protein in HCC827 compared to H23 cell line. (C) Immunoblots depict the SOX2 protein expression levels in HCC827 cells with gMock or gSOX2. (D) No alteration of the long-term growth rate by *SOX2* knockout in HCC827 cells. The representative images (left) and the corresponding quantification (right) illustrate the relative colony numbers in each sample. (E) *SOX2* knockout does not affect the formation of tumor spheres in HCC827 cells. The representative images (top) and the quantification of tumor sphere numbers (bottom) are presented. ns, not significant. Scale bar: 100 μm. (F) *SOX2* knockout does not suppress the invasion of HCC827 cells. The representative images (left) and the quantification of invaded cells (right) are provided. Mean ± SD from at least three independent experiments. Scale bar: 100 μm. **, P < 0.01.

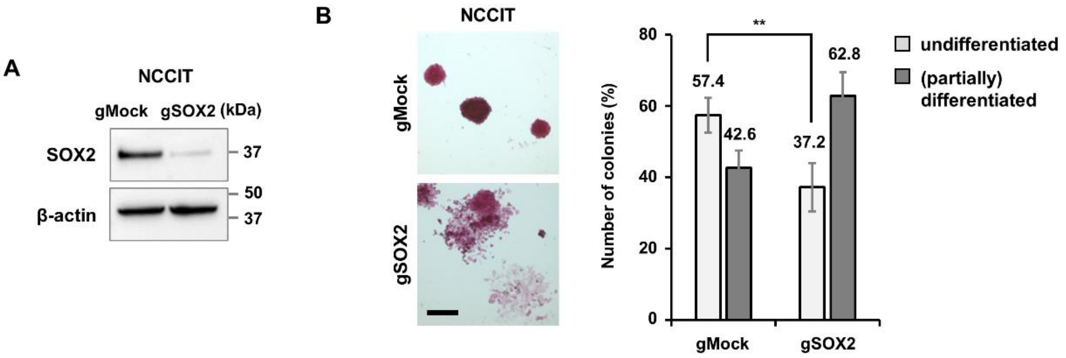

Figure S2. ***SOX2*** **knockout reduces self-renewal ability of embryonic carcinoma cells, NCCIT.** (A) Immunoblots for SOX2 protein expression levels in NCCIT cells with gMock or gSOX2. (B) Alkaline phosphatase (AP) assay was performed in NCCIT cells with gMock or gSOX2. Representative images are shown and relative colony numbers are expressed as percentages (n = 3). **, P < 0.01. Scale bar: 100 μm.
